# Coronavirus Disease Outbreak in Call Center, South Korea

**DOI:** 10.3201/eid2608.201274

**Published:** 2020-08

**Authors:** Shin Young Park, Young-Man Kim, Seonju Yi, Sangeun Lee, Baeg-Ju Na, Chang Bo Kim, Jung-il Kim, Hea Sook Kim, Young Bok Kim, Yoojin Park, In Sil Huh, Hye Kyung Kim, Hyung Jun Yoon, Hanaram Jang, Kyungnam Kim, Yeonhwa Chang, Inhye Kim, Hyeyoung Lee, Jin Gwack, Seong Sun Kim, Miyoung Kim, Sanghui Kweon, Young June Choe, Ok Park, Young Joon Park, Eun Kyeong Jeong

**Affiliations:** Korea Centers for Disease Control and Prevention, Cheongju, South Korea (S.Y. Park, Y.-M. Kim, S. Yi, S. Lee, H. Lee, J. Gwack, S.S. Kim, M. Kim, S. Kweon, O. Park, Y.J. Park, E.K. Jeong);; Seoul Metropolitan Government, Seoul, South Korea (B.-J. Na, J.-i. Kim, H.S. Kim, Y.B. Kim);; Seoul Health Foundation, Seoul (C.B. Kim);; Seoul Center for Infectious Disease Control and Prevention, Seoul (Y. Park, I.S. Huh);; Incheon Metropolitan City, Incheon, South Korea (H.K. Kim, H.J. Yoon, H. Jang);; Gyeonggi Provincial Office, Suwon, South Korea (K. Kim, Y. Chang, I. Kim);; Hallym University College of Medicine, Chuncheon, South Korea (Y.J. Choe)

**Keywords:** COVID-19, 2019 novel coronavirus disease, coronavirus disease, SARS-CoV-2, severe acute respiratory syndrome coronavirus 2, viruses, respiratory infections, zoonoses, call center, South Korea

## Abstract

We describe the epidemiology of a coronavirus disease (COVID-19) outbreak in a call center in South Korea. We obtained information on demographic characteristics by using standardized epidemiologic investigation forms. We performed descriptive analyses and reported the results as frequencies and proportions for categoric variables. Of 1,143 persons who were tested for COVID-19, a total of 97 (8.5%, 95% CI 7.0%–10.3%) had confirmed cases. Of these, 94 were working in an 11th-floor call center with 216 employees, translating to an attack rate of 43.5% (95% CI 36.9%–50.4%). The household secondary attack rate among symptomatic case-patients was 16.2% (95% CI 11.6%– 22.0%). Of the 97 persons with confirmed COVID-19, only 4 (1.9%) remained asymptomatic within 14 days of quarantine, and none of their household contacts acquired secondary infections. Extensive contact tracing, testing all contacts, and early quarantine blocked further transmission and might be effective for containing rapid outbreaks in crowded work settings.

Since the first imported case of coronavirus disease (COVID-19) was confirmed in South Korea on January 20, 2020, a sharp increase in the number of COVID-19 cases has been observed, with most infections being reported from specific clusters (*1*). Outbreaks of COVID-19 related to mass gathering, religious activities, workplaces, and hospitals have accounted for the largest portion cases in the national outbreak (*1*).

In March 2020, the Korea Centers for Disease Control and Prevention (KCDC), South Korea’s national-level public health authority, was informed about a cluster of cases of COVID-19 in a call center located in a commercial–residential mixed-use building (building X) in the capital city of Seoul. We describe the epidemiology of this COVID-19 outbreak and detail the containment efforts to limit the spread of the disease.

## Materials and Methods

### Setting

On March 8, the Seoul Metropolitan Government was notified of a confirmed case of COVID-19 in a person who worked in building X; the case reportedly was associated with a possible cluster of cases. On March 9, KCDC and local governments (in Seoul, the city of Incheon, and Gyeonggi Province) formed a joint response team and launched an epidemiologic investigation with contact tracing. Building X is a 19-story floor in one of the busiest urban area of Seoul. Commercial offices are located on the 1st through 11th floors, and residential apartments are located on the 13th through 19th floors. We identified and investigated 922 employees who worked in the commercial offices, 203 residents who lived in the residential apartments, and 20 visitors. The call center is located on the 7th through 9th floors and the 11th floor; it has a total of 811 employees. Employees do not generally go between floors, and they do not have an in-house restaurant for meals.

### Case Definition

We defined a patient under investigation (PUI) as one who worked at, lived at, or visited building X during February 21–March 8, 2020. We defined a confirmed case-patient as a PUI with a positive COVID-19 laboratory test. We confirmed the diagnosis of COVID-19 by using real-time reverse transcription PCR assays. We defined a symptomatic case-patient as a confirmed case-patient with symptoms at the time of positive testing, a presymptomatic case-patient as a confirmed case-patient who was asymptomatic at the time of positive testing but later had symptoms during the 14 days of monitoring, and an asymptomatic case-patients as confirmed a case-patient with a positive COVID-19 test result who remained asymptomatic during the entire 14-day period.

### Response Measures

Building X was closed on March 9, 2020, immediately after the outbreak was reported. We offered testing to all occupants (office workers and apartment residents) during March 9–12. We collected nasopharyngeal and oropharyngeal swab specimens from PUIs for immediate real-time reverse transcription PCR testing; the average turnaround time was 12–24 hours. Confirmed case-patients were isolated, and negative case-patients were mandated to stay quarantined for 14 days. We followed and retested all test-negative case-patients until the end of quarantine. We also investigated, tested, and monitored household contacts of all confirmed case-patients for 14 days after discovery, regardless of symptoms. During March 13–16, we sent a total of 16,628 text messages to persons who stayed >5 minutes near the building X; we tracked these persons by using cell phone location data. The messages instructed the recipients to avoid contact with others and go to the nearest COVID-19 screening center to get tested.

### Data Collection and Analysis

We obtained information on demographic characteristics and presence of symptoms through face-to-face interviews with case-patients, using standardized epidemiologic investigation forms. We performed descriptive analyses reported the results as frequencies and proportions for categoric variables. The investigation was a part of public health response and was not considered research subject to institutional review board approval; therefore, written informed consent by participants was not required.

## Results

**Figure 1:**
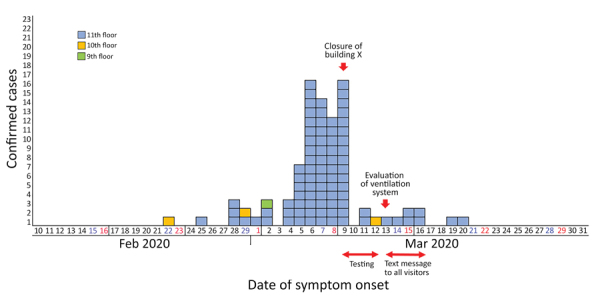
Epidemic curve of a coronavirus disease outbreak in a call center, by date of symptom onset, Seoul, Korea, 2020. Asymptomatic cases are excluded.

**Figure 2:**
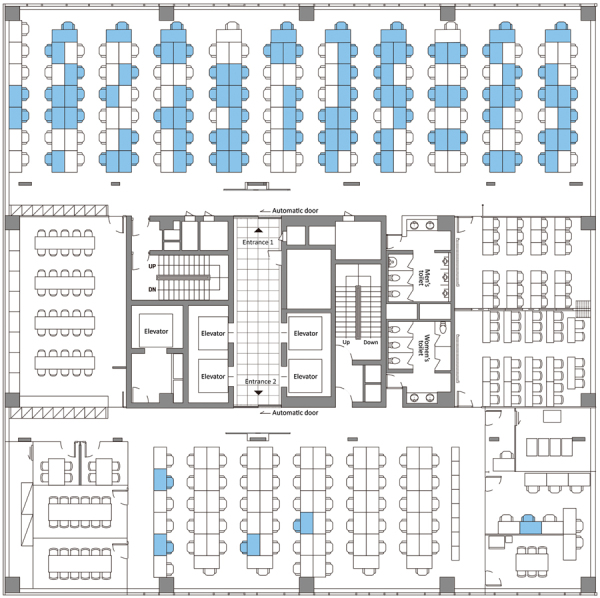
Floor plan of the 11th floor of building X, site of a coronavirus disease outbreak, Seoul, South Korea, 2020. Blue indicates the seating places of persons with confirmed cases.

Of 1,145 PUIs, we tested 1,143 (99.8%) for COVID-19 (922 employees, 201 residents, and 20 visitors) and identified 97 (8.5%, 95% CI 7.0–10.3) confirmed case-patients (Figure 1). Of 857 patients for whom demographic information was available, 620 (72.3%) were women; mean age was 38 years (range 20–80 years). Most (94 [96.9%]) of the confirmed case-patients were working on the 11th-floor call center, which had a total of 216 employees, resulting in an attack rate of 43.5% (95% CI 36.9%–50.4%) (Table 1; Figure 2). Most of the case-patients on the 11th floor were on the same side of the building. Among the 97 confirmed case-patients, 89 (91.7%) were symptomatic at the time of investigation and 4 (4.1%) were presymptomatic during the time of investigation but later had onset of symptoms within 14 days of monitoring; 4 (4.1%) case-patients remained asymptomatic after 14 days of isolation.

**Table 1:** Attack rate by location during a coronavirus disease outbreak in a call center, Seoul, South Korea, 2020

Location type and floor	Potentially exposed, no. (%)	Confirmed cases, no. (%)	Attack rate, % (95% CI)
Commercial			
1st–6th	84 (7.3)	0	0
7th (call center)	182 (15.9)	0	0
8th (call center)	207 (18.1)	0	0
9th (call center)	206 (18.0)	1 (1.0)	0.5 (0.0–3.1)
10th	27 (2.4)	2 (2.1)	7.4 (1.3–25.8)
11th (call center)	216 (18.9)	94 (96.9)	43.5 (36.9–50.4)
Residential			
13th–19th	201 (17.6)	0	0
Other	20 (1.7)	0	0
Total	1,143	97	8.5 (7.0–10.3)

The first case-patient with symptom onset, who worked in an office on the 10th floor (and reportedly never went to 11th floor), had onset of symptoms on February 22. The second case-patient with symptom onset, who worked at the call center on the 11th floor, had onset of symptoms on February 25. Residents and employees in building X had frequent contact in the lobby or elevators. We were not able to trace back the index case-patient to another cluster or an imported case.

We followed up on a total of 225 household contacts of confirmed COVID-19 case-patients (average 2.3 household members per confirmed case-patient). COVID-19 had occurred in 34 household members who had contact with symptomatic case-patients, translating to a secondary attack rate of 16.2% (Table 2). Among 11 household members of presymptomatic case-patients and 4 household members of asymptomatic case-patients, none had COVID-19 symptoms nor tested positive after 14 days of quarantine.

**Table 2:** Household secondary attack rate, by presence of symptoms, during a coronavirus disease outbreak in in a call center, Seoul, South Korea, 2020

Symptom status of index patients	Exposed, no. (%)	Confirmed cases, no. (%)	Secondary attack rate, % (95% CI)
Symptomatic	210 (93.3)	34 (100.0)	16.2 (11.6–22.0)
Presymptomatic	11 (4.8)	0	0
Asymptomatic	4 (1.9)	0	0
Total	225	34	15.1 (10.8–20.6)

## Discussion

We described the epidemiologic characteristics of a COVID-19 outbreak centered in a call center in South Korea. We identified 97 confirmed COVID-19 case-patients in building X, indicating an attack rate of 8.5%. However, if we restrict our results the 11th floor, the attack rate was as high as 43.5%. This outbreak shows alarmingly that severe acute respiratory syndrome coronavirus 2 (SARS-CoV-2) can be exceptionally contagious in crowded office settings such as a call center. The magnitude of the outbreak illustrates how a high-density work environment can become a high-risk site for the spread of COVID-19 and potentially a source of further transmission. Nearly all the case-patients were on one side of the building on 11th floor. Severe acute respiratory syndrome coronavirus, the predecessor of SARS-CoV-2, exhibited multiple superspreading events in 2002 and 2003, in which a few persons infected others, resulting in many secondary cases. Despite considerable interaction between workers on different floors of building X in the elevators and lobby, spread of COVID-19 was limited almost exclusively to the 11th floor, which indicates that the duration of interaction (or contact) was likely the main facilitator for further spreading of SARS-CoV-2.

Unique features of this outbreak investigation include a complete 14-day follow-up of close contacts of case-patients after containment measures were implemented. Close contact with an infected person is a well-recognized risk factor for acquiring SARS-CoV-2 (*2*). In recent a US study, the symptomatic secondary attack rate among 445 close contacts of COVID-19 case-patients was 10.5% among household members (*3*). In this outbreak in South Korea, we found that the secondary attack rate within the household was 16.5% among symptomatic index case-patients, which is consistent with other reports.

The role of asymptomatic COVID-19 case-patients in spreading the disease is of great concern. Among 97 confirmed COVID-19 case-patients in this study, 4 (4.1%) remained asymptomatic during the 14-days of monitoring. This rate is lower than the 30.8% rate estimated in previous modeling (*4*). A case-patient series from Beijing, China, indicated that asymptomatic case-patients accounted for 5% (13/262) of patients transferred to a designated COVID-19 hospital (*5*). Our data might represent the likely proportion of asymptomatic COVID-19 infections in the community setting. We also found that, among 17 household contacts of asymptomatic case-patients, none had secondary infections. Previous reports have postulated that SARS-CoV-2 in asymptomatic (or presymptomatic) case-patients might become transmissible to others (*6*); however, given the high degree of self-quarantine and isolation measures that were instituted after March 8 among this cohort, our analyses might have not detected the actual transmissibility in asymptomatic COVID-19 case-patients. Robust mass testing of all suspected case-patients might have prevented asymptomatic transmission because asymptomatic persons were given information about their possible infection and therefore might have self-isolated from their household members.

This outbreak investigation has several limitations. First, we could not track these cases to another cluster, making it difficult to identify the actual index case-patient. Second, not all clinical information was available for all confirmed cases, prohibiting detailed description of clinical syndromes. Date of symptom onset by office seat would be informative in understanding SARS-CoV-2 transmission in close contact area. However, our findings demonstrate the power of screening all potentially exposed persons and show that early containment can be implemented and used in the middle of national COVID-19 outbreak. By testing all potentially exposed persons and their contacts to facilitate the isolation of symptomatic and asymptomatic COVID-19 case-patients, we might have helped interrupt transmission chains. In light of the shift to a global pandemic, we recommend that public health authorities conduct active surveillance and epidemiologic investigation in this rapidly evolving landscape of COVID-19.

In summary, this outbreak exemplifies the threat posed by SARS-CoV-2 with its propensity to cause large outbreaks among persons in office workplaces. Targeted preventive strategies might help mitigate the risk for SARS-CoV-2 infection in these vulnerable groupS.
